# Preliminary Evidence for Aortopathy and an X-Linked Parent-of-Origin Effect on Aortic Valve Malformation in a Mouse Model of Turner Syndrome

**DOI:** 10.3390/jcdd2030190

**Published:** 2015-07-10

**Authors:** Robert B. Hinton, Amy M. Opoka, Obah A. Ojarikre, Lawrence S. Wilkinson, William Davies

**Affiliations:** 1 The Heart Institute, Division of Cardiology, Cincinnati Children’s Hospital Medical Center, Cincinnati, OH 45208, USA; E-Mail: amy.opoka@cchmc.org; 2 Mill Hill Laboratory, Francis Crick Institute, London NW7 1AA, UK; E-Mail: andrew.ojarikre@crick.ac.uk; 3 MRC Centre for Neuropsychiatric Genetics and Genomics and Institute of Psychological Medicine and Clinical Neurosciences, School of Medicine, Cardiff University, Cardiff CF24 4HQ, UK; E-Mails: wilkinsonl@cardiff.ac.uk (L.S.W.); daviesw4@cardiff.ac.uk (W.D.); 4 Neuroscience and Mental Health Research Institute, Schools of Medicine and Psychology, Cardiff University, Cardiff CF24 4HQ, UK

**Keywords:** cardiovascular malformation, valves, heritability, disease models, genomic imprinting, *Xlr3b*, *FAM9B*, 39,XO

## Abstract

Turner syndrome (TS), most frequently caused by X-monosomy (45,X), is characterized in part by cardiovascular abnormalities, including aortopathy and bicuspid aortic valve (BAV). There is a need for animal models that recapitulate the cardiovascular manifestations of TS. Extracellular matrix (ECM) organization and morphometrics of the aortic valve and proximal aorta were examined in adult 39,XO mice (where the parental origin of the single X was paternal (39,X^P^O) or maternal (39,X^M^O)) and 40,XX controls. Aortic valve morphology was normal (tricuspid) in all of the 39,X^P^O and 40,XX mice studied, but abnormal (bicuspid or quadricuspid) in 15% of 39,X^M^O mice. Smooth muscle cell orientation in the ascending aorta was abnormal in all 39,X^P^O and 39,X^M^O mice examined, but smooth muscle actin was decreased in 39,X^M^O mice only. Aortic dilation was present with reduced penetrance in 39,XO mice. The 39,XO mouse demonstrates aortopathy and an X-linked parent-of-origin effect on aortic valve malformation, and the candidate gene *FAM9B* is polymorphically expressed in control and diseased human aortic valves. The 39,XO mouse model may be valuable for examining the mechanisms underlying the cardiovascular findings in TS, and suggest there are important genetic modifiers on the X chromosome that modulate risk for nonsyndromic BAV and aortopathy.

## 1. Introduction

Turner syndrome (TS), most frequently caused by X-monosomy (45,X), is characterized by diverse developmental, endocrine and cardiovascular abnormalities, and affects approximately 1 in 2500 live born females [1]. Cardiovascular malformations (CVM) are present in 75% of TS fetuses and 20%–45% of live born infants, with bicuspid aortic valve (BAV), typically resulting from fusion of the right and left coronary cusps, being the most commonly observed malformation (20%–30% of TS cases) [2,3]. Aortic coarctation and thoracic aortic aneurysm also occur [4].

Two distinct genetic mechanisms may contribute to the phenotype of individuals with a 45,X constitution: first, haploinsufficiency for one or more of products of genes that escape X-inactivation (~15%–20% of the X-linked genetic complement [5]). Second, phenotypic variability within 45,X individuals may be due to the parental origin of the single X chromosome; in ~70% of TS cases, the X chromosome is inherited from the mother (45,X^M^) whereas in the remainder it is inherited from the father (45,X^P^). X-linked parent-of-origin effects (POE), *i.e.* where the appearance of 45,X^M^ and 45X^P^ subjects differs significantly, have been described on diverse phenotypes including: brain morphology and cognitive function, neck webbing, sensorineural hearing loss, renal and ocular abnormalities [6,7,8,9]. Some limited evidence exists for increased rates of non-syndromic CVMs (including aortic valve malformation and aortopathy) in 45,X^M^ subjects relative to 45,X^P^ subjects [6,10], somewhat consistent with increased rates of aortic valve malformation in males (46,X^M^Y) relative to females (46,X^P^X^M^) [11]. However, other studies have shown no difference in CVMs, including specifically BAV, between 45,X^M^ and 45,X^P^ individuals [2,12,13]. X-linked POE could be explained by the presence of X-linked imprinted genes (*i.e*., genes that are solely, or predominantly, expressed from either the paternally or maternally inherited allele) [14]; to date, no X-linked imprinted genes have been identified in man. Alternatively, such POE might be explained by the presence of cryptic mosaicism (notably the presence of Y-linked sequences in 45,X^M^ individuals only) [15].

The 39,XO mouse model, where the parental origin of the X chromosome can be varied (39,X^P^O or 39,X^M^O) partially recapitulates several aspects of the Turner syndrome phenotype, including X-linked POE on cognition [16]; as a consequence of how they are generated, these mice cannot possess cryptic X or Y-linked sequences [17]. Here, we studied the cardiovascular system of 39,XO mice for the first time. We hypothesized that 39,X^M^O mice would exhibit higher rates of aortopathy and aortic valve malformation than 39,X^P^O (and 40,XX female) mice.

## 2. Methods and Materials

40,XX, 39,X^P^O and 39,X^M^O mice were bred on an MF1 outbred albino strain background and karyotyped *post mortem* as described previously [16]. Mice aged 6 weeks to 12 months were used. We used an outbred background because: (a) 39,XO mouse physiology has previously been extensively characterized on this background; and (b) humans are not inbred, hence the data obtained in our model may have greater translational relevance. Adult hearts were dissected, fixed in 4% paraformaldehyde, dehydrated in ethanol and stored in 70% ethanol at −20 °C before histological processing. Importantly, cardiovascular morphology was analyzed blind to karyotype. Briefly, short axis serial sections showing the aortic valve *en face* were examined to determine aortic valve morphology based on the number and location of cusp attachments, which was classified as unicuspid, bicuspid, tricuspid (normal), or quadricuspid according to established definitions [18]. Morphometrics were performed using sections obtained in the long axis to examine aortic valve tissue (annulus area, cusp length, and hinge thickness), aorta tissue (medial thickness in the aortic root and ascending aorta), and the caliber of the aortic lumen at different levels (aortic valve annulus, aortic root, sinotubular junction and ascending aorta dimensions), analogous to clinical measures, as previously described [19]. By early adulthood the bodyweights of 40,XX and 39,XO mice do not differ significantly [16], therefore there is no need to normalize aorta measurements for body size as is done in TS subjects due to short stature [20,21]. Extracellular matrix (ECM) organization of both the aortic valve and the ascending aorta was assessed using Movat’s pentachrome stain [22]. The aorta was studied further by examining the presence of alpha smooth muscle actin (SMA) and phospho-histone H3 (a marker of proliferation) using immunohistochemistry. Smooth muscle cell orientation was assessed by measuring the angle of the plane of the cell relative to the adjacent elastic fiber; an angle greater than 60 degree was considered abnormal. Our model allowed us to assess the effects of X-monosomy (40,XX *vs.* 39,XO), and X-linked POE (39,X^P^O *vs.* 39,X^M^O) on the measures described. Morphometric data were analyzed by One Way ANOVA with a between-group factor of KARYOTYPE. Data regarding frequency of aortic valve malformations were analyzed by a one-tailed Freeman Halton extension of Fisher’s Exact Test. Human *FAM9B* expression was examined in human aortic valve control and disease samples by reverse transcriptase polymerase chain reaction using the following primers: 5′-CGCCTCTAGCTTCCCAGGACAA-3′ and 5′-GGCTTCTTGGTATTAGCCCCCGT-3′. *FAM9B* cDNA was used as the positive control (Thermo Fisher MGC cDNA, MHS6278-211690668).

## 3. Results and Discussion

### 3.1. Aortic Valve

**Figure 1:**
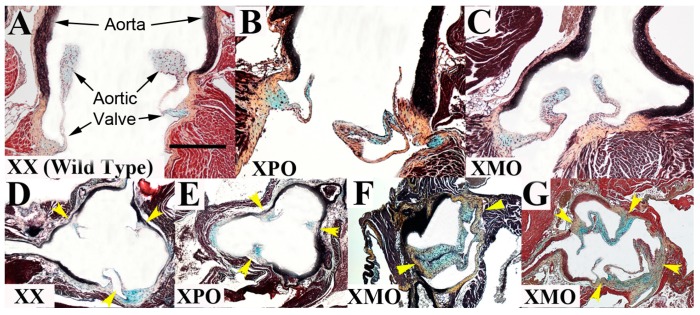
Aortic valve morphology is abnormal in 39,X^M^O mice only. Aortic valves and proximal aortas are shown in the long axis view in adult 40,XX wild type controls (**A**) 39,X^P^O (**B**) and 39,X^M^O (**C**) mice. Trilaminar extracellular matrix (ECM) organization in both aortic valve and aorta tissue is preserved in 39, XO mice (**B**,**C**). Normal aortic valve morphology is seen in the short axis view in 40,XX (**D**) and 39,X^P^O mice (**E**), *i.e.*, a tricuspid pattern with three commissures (yellow arrowheads); however, 39,X^M^O mice demonstrate a variety of malformation patterns, including bicuspid aortic valve (BAV), as evidenced by two commissures (**F**), and quadricuspid aortic valve, which shows four commissures (**G**). Panels shown at 20× magnification; scale bar 250 μm.

The overall prevalence of aortic valve malformation in 39,XO mice was 4/45 (8%). While aortic valve morphology was normal (tricuspid) in all of the 39,X^P^O (*n* = 18) and 40,XX (*n* = 21) mice studied, four mice (15%) from the 39,X^M^O group (*n* = 27) exhibited valve abnormalities (*p* = 0.03); three 39,X^M^O mice displayed BAV and one a quadricuspid aortic valve (Figure 1D–G). Two of the BAVs seen in the 39,X^M^O group had fusion of the right and left coronary cusps, and one had fusion of the right and non-coronary cusps, consistent with the frequency identified in human studies [2]. There was no gross evidence of raphe or partial fusion. The aortic valve findings appear highly specific in that ECM trilaminar organization and valve morphometrics were equivalent in 40,XX, 39,X^P^O and 39,X^M^O mice (One Way ANOVA effect of KARYOTYPE on hinge thickness: F_[2,23]_ = 1.58, *p* = 0.23, cusp thickness: F_[2,23]_ = 0.21, *p* = 0.81, cusp length: F_[2,23]_ = 0.51, *p* = 0.61 and on annulus area: F_[2,23]_ = 1.19, *p* = 0.32, Table 1, Figure 1). While our current data provide intriguing initial support for an X-linked parent-of-origin effect on aortic valve malformation, we cannot completely discount the possibility that such abnormalities are seen in both 39,X^P^O and 39,X^M^O groups; this will require testing in further larger-scale studies.

**Table 1:** Aortic Valve and Aorta Morphometrics in a Mouse Model of Turner Syndrome.

	Valve Annulus Area (μm^2^)	Valve Hinge Thickness (μm)	Valve Cusp Length (μm)	Aortic Root Thickness (μm)	Ascending Aorta Thickness (μm)
XX ( *n* = 8)	5563 ± 1628	47 ± 14	279 ± 57	58 ± 8	111 ± 16
X^P^O (*n* = 6)	4738 ± 1639	50 ± 6	325 ± 101	63 ± 4	103 ± 5
X^M^O (*n* = 12)	7180 ± 1328	59 ± 19	318 ± 112	57 ± 7	104 ± 14

Mean ± Standard Deviation.

### 3.2. Ascending Aorta

**Figure 2:**
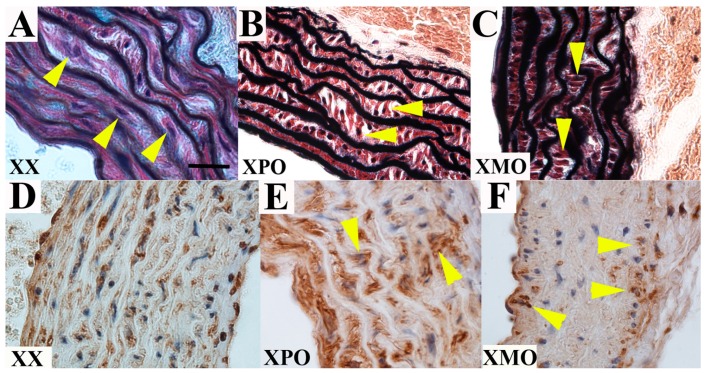
Aorta smooth muscle cell orientation is abnormal in both 39,X^M^O and 39,X^P^O mice, but content is decreased in 39,X^M^O mice only. High magnification of the ascending aorta in adult 40,XX wild type controls (**A**) 39,X^P^O (**B**) and 39,X^M^O (**C**) mice demonstrate intact elastic fiber architecture and subtle smooth muscle cell misalignment abnormalities in both 39,X^P^O and 39,X^M^O mice. Note the plane of the smooth muscle cells in the 39, XO mice (yellow arrowheads) is orthogonal to the plane of elastic fibers, when compared with the normal parallel orientation of the cells (yellow arrowheads in panel H). SMA staining shows normal diffuse expression in 40,XX (**D**) aortas, as well as 39,X^P^O (**E**) aortas despite the cell orientation abnormality, but significantly decreased expression in 39,X^M^O mice (**F**). Panels are shown at 100×; scale bar 25 μm.

There were no significant differences across 40,XX (*n* = 5), 39,X^P^O (*n* = 5) and 39,X^M^O (*n* = 9) groups with regard to aortic root dimensions (F_[2,16]_ = 3.14, *p* = 0.07), aortic dimensions (F_[2,14]_ = 2.38, *p* = 0.13), or aortic valve annulus size (F_[2,16]_ = 2.08, *p* = 0.16), but there appeared to be a trend whereby all aortic measurements were greater in 39,XO mice than 40,XX mice with 39,X^M^O mice showing the greatest deviation from 40,XX subjects; a proportion of 39,XO mice showed aorta measures greater than two standard deviations from the 40,XX mean (Table 2). Interestingly, while elastic fiber architecture, including gross lamellae number and medial thickness, was ostensibly normal across all three experimental groups, smooth muscle cell orientation in the ascending aorta was abnormal in all 39,X^P^O and 39,X^M^O mice examined when compared with 40,XX mice, demonstrating misaligned cells arranged in a plane orthogonal to the lamellae rather than the normal parallel plane; the average proportion of cells misaligned in 40,XX (*n* = 5), 39,X^P^O (*n* = 5) and 39,X^M^O (*n* = 9) was 12.5%, 72.7% and 82.6%, respectively (*x*^2^_[2]_ = 165.43, *p* < 0.0001, Figure 2A–C). The cell density and proliferation index were not different between genotypes (data not shown). Interestingly, SMA staining was decreased in 39,X^M^O mice only (Figure 2D–F). To our knowledge, this intriguing phenotype has not previously been reported in individuals with TS and warrants further investigation in this population.

**Table 2:** Aorta Dimensions by Pathology in a Mouse Model of Turner Syndrome.

	Valve Annulus Dimension (μm)	Aortic Root Dimension (μm)	Sinotubular Junction Dimension (μm)	Ascending Aorta Dimension (μm)
XX (*n* = 5)	486 ± 40	735 ± 52	502 ± 54	517 ± 41
X^P^O (*n* = 5)	500 ± 36	778 ± 65	531 ± 62	559 ± 25
	(0/5, 0%)	(1/5, 20%)	(1/5, 20%)	(1/5, 20%)
X^M^O (*n* = 9)	537 ± 50	826 ± 63	540 ± 59	566 ± 44
	(3/9, 33%)	(5/9, 56%)	(3/9, 33%)	(2/7, 29%)

Mean ± Standard Deviation; parentheses reflect proportion of mice > 2 SDs from the XX mean.

### 3.3. Discussion

Here we have demonstrated preliminary evidence for aortic valve malformation and aortopathy in the 39,XO mouse. Our aortic data from mutant mice suggest abnormalities in smooth muscle cell adhesion to the elastic fiber, similar to previous studies in a mouse model of aortopathy [23], but not overt elastic fiber pathology, and a propensity for latent dilation of the proximal aorta, particularly in 39,X^M^O subjects. Aortic dilation in 39,XO mice is consistent with observations in TS individuals with aortic valve pathology [24]; unfortunately as our aortic valve and aortic size measurements were assessed from short and long axis sections respectively, we could not determine the frequency of both defects being present in the same individual mice. Further *in vivo* work in large cohorts of older 39,XO animals, as well as replication in other inbred strains, will be required to identify mechanisms underlying the aberrant smooth muscle orientation observation, to verify any dilated aorta phenotype, and to more accurately ascertain its prevalence, age of onset, potential POEs and relevance to pathophysiology. While the variable ages used in this study do not impact the evaluation of a congenital defect like BAV, they do confound the determination of TAA since the manifestation of this disease is typically sometime after birth. In addition, a more comprehensive assessment of cardiac structure in these mice is warranted to evaluate for subtle but clinically significant CVM associated with TS, such as partial anomalous pulmonary venous return. The finding of a quadricuspid aortic valve suggests there is a related spectrum of aortic valve malformation ranging from unicuspid to quadricuspid, a concept that has been forwarded previously [25]. While there was no conspicuous evidence of more severe CVM, such as hypoplastic left heart syndrome (HLHS), it is important to note that 39,XO mice, unlike TS patients, do not appear to show significant *in utero* mortality, an observation that may represent a significant limitation of the model but also suggests this mouse may be a good model for individuals with TS who survive to birth, including those who may be XX, XO mosaics. Lymphatic abnormalities resulting in hydrops are widely regarded as the primary cause of fetal loss in TS. Studies have sought to establish that “flow-related” CVMs, ranging from BAV to HLHS are caused by the hygroma in TS, but CVM occurs without lymphatic abnormality, fetal loss occurs in the absence of CVM, and severe CVM, including HLHS, is viable in TS [26,27], suggesting that CVM is neither necessary nor sufficient for fetal loss in TS. The lack of fetal loss in mice may be attributable to the fact that far fewer X-linked genes escape X-inactivation in the mouse than in humans (hence the loss of an X chromosome is less deleterious).

Our aortic valve malformation data indicate an overall prevalence of ~8% in 39,XO mice (consistent with increased rates in TS) with 39,X^M^O mice being selectively affected. While this putative X-linked POE on aortic valve morphology cannot be explained by cryptic mosaicism, it could theoretically result from the fact that the 39,X^M^O mice are generated from a separate cross to 40,XX and 39,X^P^O mice; the fact that the 39,X^M^O mice were born to wild type 40,XX mothers, and raised in identical conditions to the other two experimental groups strongly argues against this possibility. The most parsimonious explanation for this finding is that there are one or more X-linked imprinted genes that influence aortic valve development in mice; the expression/function of these genes might be affected by modifier genes and/or the environment, hence why not all 39,X^M^O mice exhibited aortic valve abnormalities. To date, the only X-linked gene known to be imprinted in the mouse heart is *Xlr3b*, the expression of which is approximately 12-fold greater in 39,X^M^O than 39,X^P^O mice during late embryogenesis [16]. The function(s) of *Xlr3b* are currently unknown, but roles in chromatin metabolism and in response to toxic insults have been suggested [14,28]. Therefore, *Xlr3b* represents a potentially important genetic candidate for predisposing to developmental aortic valve abnormalities in mice, and further work on the role of this protein within the mammalian heart is warranted. It will also be worthwhile comparing gene expression in 39,X^P^O and 39,X^M^O heart tissue to identify novel underlying X-linked imprinted gene candidates.

**Figure 3:**
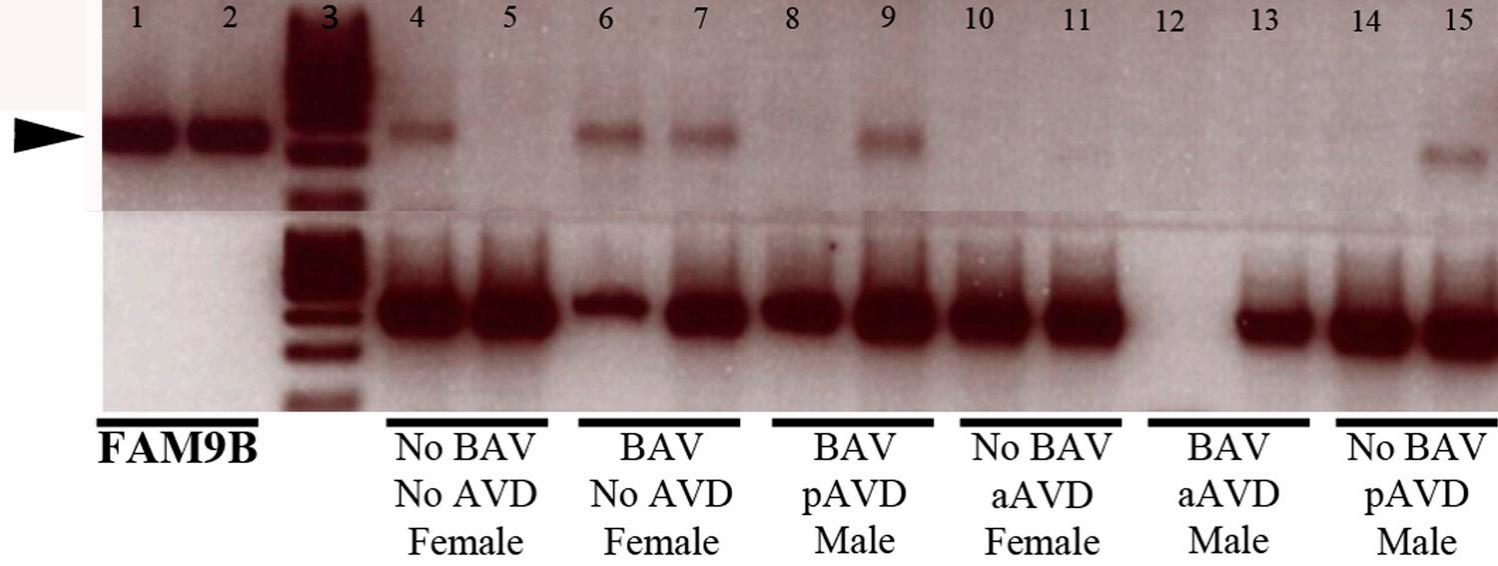
*FAM9B* is polymorphically expressed in human aortic valve tissue. *FAM9B* is expressed in control (No BAV, No AVD) human aortic valve tissue; however, there is no clear relationship between *FAM9B* expression (black arrowhead at ~275 bp) status, when compared with positive control expression from a human *FAM9B* cDNA clone (left columns), and aortic valve morphology (tricuspid or bicuspid), the presence of aortic valve disease (pediatric or adult), or gender (male or female). Samples were run simultaneously two per group (black bars), except BAV aAVD Male due to a technical failure (lane 12), and beta-actin (ACTB) was used as the housekeeping gene. BAV bicuspid aortic valve; AVD aortic valve disease; p: pediatric; a: adult; loading control (lane 3).

In terms of relevance to Turner syndrome, our data indicate two possibilities: (i) there is an X-linked POE on aortic valve morphology in mice but not in humans due to species’ different physiology; or (ii) there is an X-linked POE in both species, in the same direction, and of a similar magnitude. It is possible that, in some studies, an X-linked POE in humans could have been obscured by the presence of mosaicism (*i.e*., 46,XX and 45,X^M^O cells in some individuals), by the relatively low frequency of abnormalities in the 45,X^M^ group, and/or by using scanning techniques to examine cardiac morphology rather than *post mortem* histology. An obvious candidate gene for any X-linked POE on aortic valve development in humans is *FAM9B*, the closest orthologue of *Xlr3b* [16]. *FAM9B* is located on the short arm of the X chromosome at Xp22.32 within a region recently shown to be important in aortic valve and aorta development [29]. In an additional analysis, we have shown that *FAM9B* is polymorphically expressed in human aortic valves (Figure 3). In this small sample, there was no clear relationship between *FAM9B* expression status and gender (male or female), aortic valve morphology (tricuspid or bicuspid), or the presence of aortic valve disease (AVD) (pediatric or adult). Whether *FAM9B* is imprinted in human heart tissue, why it is polymorphically expressed in the aortic valve, and how it influences cardiac biology remains to be investigated.

## 4. Conclusions

In summary, our findings suggest the 39,XO mouse as a much-needed animal model for examination of the biological mechanisms underlying the cardiovascular findings in Turner syndrome, and indicate that there may be important genetic modifiers on the X chromosome in nonsyndromic BAV, aortic valve disease and aortopathy. Specifically, our work suggests *FAM9B* as a potential novel X-linked genetic modulator of CVM risk.
